# Effective inhibition of MYC-amplified group 3 medulloblastoma by FACT-targeted curaxin drug CBL0137

**DOI:** 10.1038/s41419-020-03201-6

**Published:** 2020-12-02

**Authors:** Jiajia Wang, Yi Sui, Qifeng Li, Yang Zhao, Xiaoshu Dong, Jian Yang, Zhuangzhuang Liang, Yipeng Han, Yujie Tang, Jie Ma

**Affiliations:** 1 Department of Pediatric Neurosurgery, Xinhua Hospital, Shanghai Jiao Tong University School of Medicine, Shanghai, People’s Republic of China; 2 Key Laboratory of Cell Differentiation and Apoptosis of National Ministry of Education, Department of Pathophysiology, Shanghai Jiao Tong University School of Medicine, Shanghai, People’s Republic of China

**Keywords:** Target validation, CNS cancer

## Abstract

Medulloblastoma (MB) is the most common malignant pediatric brain tumor that can be categorized into four major molecular subgroups. Group 3 MB with *MYC* amplification (MYCamp-G3-MB) has been shown to be highly aggressive and exhibited worst prognosis, indicating the need for novel effective therapy most urgently. A few epigenetic targeted therapeutic strategies have recently been proven to effectively treat preclinical models of MYCamp-G3-MB, including BET inhibition, HDAC inhibition and SETD8 inhibition, unveiling a promising direction for further investigation. In this study, we carried out systemic bioinformatic analyses of public-available MB datasets as well as functional genomic screening datasets of primary MYCamp-G3-MB lines to search for other potential therapeutic targets within epigenetic modulators. We identified SSRP1, a subunit of histone-chaperone FACT complex, to be the top drug target candidate as it is highly cancer-dependent in whole-genome CRISPR-Cas9 screening across multiple MYCamp-G3-MB lines; significantly upregulated in MYCamp-G3-MB compared to normal cerebellum and most of the rest MB subtypes; its higher expression is correlated with worse prognosis; and it has a blood-brain-barrier penetrable targeted drug that has entered early phase human clinical trials already. Then we utilized RNA-interference approach to verify the cancer-dependency of SSRP1 in multiple MYCamp-G3-MB lines and further confirmed the therapeutic efficacy of FACT-targeted curaxin drug CBL0137 on treating preclinical models of MYCamp-G3-MB in vitro and in vivo, including an orthotopic intracranial xenograft model. Mechanistically, transcriptome analyses showed CBL0137 preferentially suppressed cell-cycle and DNA-repair related biological processes. Moreover, it selectively disrupted transcription of *MYC* and *NEUROD1*, two critical oncogenic transcription factors of MYCamp-G3-MB, via depleting FACT complex from their promoter regions. In summary, our study demonstrates FACT-targeted CBL0137 works effectively on treating MYCamp-G3-MB, presenting another promising epigenetic-targeted therapeutic strategy against the most devastating form of MB.

## Introduction

As the most common malignant pediatric brain tumor, medulloblastoma (MB) is highly heterogeneous and consists of four major molecular subgroups including Sonic Hedgehog (SHH), Wingless (WNT), group 3 (G3), and group 4 (G4)^1,2^. Each MB subgroup manifests distinct demographic, pathological, and clinical characteristics, such as age, gender, cell of origin, oncogenic driver, tendency of metastasis, response to conventional therapy and prognosis. Among the four subgroups, group 3 medulloblastoma (G3-MB) exhibits the worst prognosis and therefore needs to identify novel effective therapy most urgently. Notably, G3-MB has been shown to be significantly associated with *MYC* amplification, which indicates an extremely poor prognosis^3,4^. The dismal outcome is associated with the high rate of metastasis and recurrence and the late diagnosis at the advanced stage of the malignancy^5^. Worse still, while chemotherapy and radiation regimens prove less efficacious in patients with MYC-amplified G3-MB (MYCamp-G3-MB), further intensification of chemotherapy or radiation could exacerbate the adverse systemic toxicity^6^.

Researchers have been making a great amount of effort to developing targeted therapies for treating MYCamp-G3-MB in recent years. Of note, multiple epigenetic modulators have been demonstrated to effective therapeutic targets, such as BRD4, HDACs, and SETD8^7–9^. Targeting BRD4 with BET inhibitor results in transcriptional inhibition of *MYC*, which is the oncogenic driver and crucial cancer-dependent gene of MYCamp-G3-MB^7,10^. HDAC inhibition attenuates growth of MYCamp-G3-MB in part by inducing expression of the *FOXO1* tumor suppressor gene^9^. SETD8 inhibition targets H4K20me chromatin occupancy at key genes involved in tumor invasiveness and pluripotency of MYCamp-G3-MB^8^. Together, these studies demonstrate that epigenetic targeted therapy represents a promising avenue for developing novel effective therapy against MYCamp-G3-MB.

SSRP1 and SPT16 constitute a heterodimer histone-chaperone complex called facilitates chromatin transcription (FACT), which can promote transcriptional elongation by facilitating disassembly and reassembly of nucleosomes^11^. It also regulates DNA replication and DNA repair. FACT complex is upregulated in many cancer types and plays a crucial role in tumorigenesis. Curaxin CBL0137, a FACT-targeted small-molecule antagonist, has been shown to exhibit broad antitumor activity. It exerts its inhibitory effect through altering DNA architecture and trapping FACT complex in chromatin without inducing DNA chemical modification^12^. The trapping could then deplete FACT from transcriptional active regions of various oncogenes in different cancer types and subsequently block their transcription^13^. More importantly, CBL0137 has entered into Phase I clinical trials on adult patients with hematological malignancies and solid tumors (ClinicalTrials.gov Identifier: NCT01905228, NCT02931110, and NCT03727789). And it is able to effectively infiltrate through the blood-brain barrier (BBB) and thereby suitable for treating brain tumors^14^.

In the present study, we carried out systemic bioinformatic analyses of public-available MB tumor datasets as well as whole-genome functional genomic screening data of patient-derived primary tumor lines, and identified SSRP1 as the top drug target candidate among epigenetic modulators in MYCamp-G3-MB. We then confirmed its cancer-dependency and further revealed SSRP1 inhibition with CBL0137 as another effective epigenetic-targeted therapeutic strategy against MYCamp-G3-MB.

## Materials and methods

### Bioinformatics analysis

#### MB tumor gene expression datasets

Medulloblastoma tumor gene expression datasets were obtained from R2 Genomics Analysis and Visualization Platform (http://r2.amc.nl, referenced accessions: Pomeroy dataset^15^, u133p2dataset (Pfister dataset^16^ + Gilbertson dataset^17^ + normal cerebellum Roth dataset^18^)) and GEO (GSE85217^19^ and GSE22875^20^) for the data analysis in the current study.

### Survival analysis

The survival analyses with respect to SSRP1 and SUPT16H expression in medulloblastoma patients were performed using a separate cohort of 612 samples from Cavalli dataset (GSE85217, 763 samples). The medulloblastoma patients were divided into low- and high-expression groups based on the median expression level. To evaluate the difference in survival time between the high-expression and low-expression medulloblastoma groups, we used log-rank tests and plotted Kaplan–Meier survival curves.

#### The effect of SSRP1 knockout on the growth of medulloblastoma cell lines

The effects of SSRP1 and SUPT16H depletion on medulloblastoma cell growth and viability were evaluated in including 4 medulloblastoma cell lines, based on the CRISPR-Cas9 screening DepMap (Cancer Dependency Map) dataset download from Project Achilles (https://depmap.org/portal/). A CERES score less than −0.5 indicated that cell growth or viability was inhibited after the depletion of target gene.

#### RNA extraction and real-time RT-PCR

TRIzol reagent (TR118, MRC) was used to isolate the total RNA from the cells, following the manufacturer’s instructions. SYBR Green Master (24759100, Roche) and High Capacity cDNA Reverse Transcription Kit (4368813, Thermo Fisher Scientific) were used to perform reverse transcription and qPCR assays. The Bio-Rad S1000^TM^ thermocycler (Bio-Rad, USA) was used to perform the reverse transcription reactions. An ABI 7900 Real-Time PCR instrument was used incubate the qPCR mixture at 95 °C for 3 min as the initial denaturation step, followed by 40 PCR cycles (95 °C for 5 s, 60 °C for 20 s, and 72 °C for 20 s). The 2^−ΔΔCq^ method was used to calculate the expression of the corresponding relative mRNA, and the relative mRNA expression level was normalized to the expression of GAPDH. The results of qPCR represent three independent experiments. The qPCR primer sequences used in the current study are listed as follows: SSRP1, 5′-TTGAGAGGGAGGAGTACGGG-3′ (forward); 5′-CTAGCTTGGGTTCATGCCCT-3′ (reverse); GAPDH, 5′-TGACTTCAACAGCGACACCCA-3′ (forward); 5′- CACCCTGTTGCTG TAGCCAAA-3′ (reverse); NEUROD1, 5′-GGTGCCTTGCTATTCTAAGACGC-3′ (forward); 5′-GCAAAGCGTCTGAACGAAGGAG-3′ (reverse); MYC, 5′-AGAGTCTGGATC ACCTTCT GCT-3′ (forward); 5′-ACACTGTCCAACTTGACCCTCT-3′ (reverse).

#### Cellular protein fractionation

Cytoplasmic and nuclear extracts were prepared as following: medulloblastoma cells were swollen in buffer A (10 mM Tris-HCl, pH 7.5, 10 mM KCl, 1.5 mM MgCl_2_, 0.34 M sucrose, 10% glycerol, 1 mM DTT, and protease inhibitors) with 0.2% Triton X-100 on ice for 10 min. Nuclei were collected in pellet by low-speed centrifugation (5 min, 1300×*g*, 4 °C). Nuclei were washed once in buffer A, and then lysed in buffer B (50 mM Tris-HCl, pH 8, 25% glycerol, 420 mM NaCl, 1.5 mM MgCl_2_, 0.2 mM EDTA, 1 mM DTT, and protease inhibitors). After 30 min on ice, insoluble proteins were removed from the nuclear extract by high-speed centrifugation (15 min, 13,000 rpm, and 4 °C).

### Western blot analysis

Primary antibodies against SSRP1 (13421S) and MYC (5605S) were purchased from Cell Signaling Technology (MA, USA). Antitubulin primary antibody (ab6046) was purchased from Abcam (Cambridge, UK).

#### shRNA plasmid

For the generation of shRNA plasmids, double-strand oligonucleotides were annealed and cloned into the TRC2-pLKO-puro vector. The oligonucleotides of shRNA were synthesized by Sangon Biological Engineering Technology (Shanghai, China). The target oligonucleotides are as the following:

shSCR-Forward: CCGGCCTAAGGTTAAGTCGCCCTCGCTCGAGCGAGGGCGACTTAACCTTAGGTTTTTG

shSCR-Reverse: AATTCAAAAACCTAAGGTTAAGTCGCCCTCGCTCGAGCGAGGGCGACTTAACCTTAGG

shSSRP1-1-Forward: CCGGCGCTTCGATGAGATCTCCTTTCTCGAGAAAGGAGATCTCATCGAAGCGTTTTTG

shSSRP1-1-Reverse: AATTCAAAAACGCTTCGATGAGATCTCCTTTCTCGAGAAAGGAGATCTCATCGAAGCG

shSSRP1-2-Forward: CCGGGCCATGGACTTAAACTGCTTACTCGAGTAAGCAGTTTAAGTCCATGGCTTTTTG

shSSRP1-2-Reverse: AATTCAAAAAGCCATGGACTTAAACTGCTTACTCGAGTAAGCAGTTTAAGTCCATGGC.

#### Lentivirus preparation and infection

Oligonucleotides were annealed and then cloned into the TRC2-pLKO-puro plasmid to generate lentiviral shRNA plasmids. Moreover, we seeded the MB cells in the 6-well plates at the confluency of about 70%. The cells were transfected with lentiviral shRNA plasmids 24 h after seeding. The transfection was performed in Gibco Opti-MEM reduced serum medium using 1.67 μg target lentiviral vector, 1 μg psPAX2, and 0.67 μg pMD2.G and PEI max (Polysciences) transfection reagents. After 6 h of incubation, the medium was removed. The first and second viral supernatants were collected 24 h and 48 h after the transfection, respectively. A 0.45-μm membrane was used to filter the harvested viral supernatant, which was then concentrated by PEG6000, resuspended, aliquoted and stored at −80 °C. Fortyeight hours of post-infection, the target cell lines infected with lentiviral shRNA were cultured in 1.0 μg/ml puromycin-containing growth medium.

#### Cell culture

D425, D458, and HDMB03 cell lines were kind gifts from Yoon-Jae Cho (Oregon Health and Science University, USA). The D425 and D458 cell lines were cultured in Dulbecco’s modified Eagle’s medium (DMEM) supplemented with 1% penicillin–streptomycin and 10% FBS. The histological features of the primary tumors of HDMB03 showed large cell medulloblastoma, with its gene expression markers consistent with the expression pattern of MYC-amplified MB. We maintained the HDMB03 cells in culture media with Neurobasal medium supplemented with B27 (Gibco), FGF (GF003, Merck Millipore, Darmstadt, Germany), EGF (02653, Stem Cell Technologies, Vancouver, BC, Canada), Heparin (07980, Stem Cell Technologies, Vancouver, BC, Canada), and LIF (LIF1010, Merck Millipore, Darmstadt, Germany).

#### Compound treatment

CBL0137 (HY-18935A) was purchased from MedChem Express (Monmouth Junction, NJ, USA). For CBL0137 treatment in vivo, stock solution was diluted with 10% cyclodextrin at the dose of 70 mg/kg every 4 days by intravenous injection.

### In vivo experiments

#### Intracranial

In vivo efficacy experiments were performed according to the following protocols. The protocol was approved by Institutional Animal Care and Use Committee at Shanghai Jiao Tong University. We used a GFP-luciferase lentiviral expression construct to infect the HDMB03 cells to obtain the GFP-luciferase-positive cells by Fluorescence Activated Cell Sorting (FACS). The GFP-luciferase-positive cells were injected into the cerebella (2 mm posterior to the lambda suture, 1–2 mm lateral to the midline, and 2.5 mm deep) with stereotaxic guidance. Subsequently, the mice were treated with d-luciferin (75 mg/kg; Promega) and were imaged on the Xenogen IVIS2000 system (Perkin-Elmer) 72 h after the injection to confirm engraftment. The nude mice were randomly assigned into the vehicle group and CBL0137 group. Tumor growth in both two groups was observed by IVIS imaging after 0, 7, 14, 21, 28, and 35 days of treatment. Finally, log-rank test was performed to evaluate the statistical significance in the Kaplan–Meier analysis.

#### Subcutaneous

Cell suspension was mixed with an equal volume of Matrigel (BD Biosciences, 354230) and was subcutaneously injected into each side of the dorsal flank. Calliper was used to measure the tumor volumes twice per week, and the tumor volumes were calculated as length × width^2^ × 0.5.

For the immunohistochemical analysis of xenografted medulloblastomas, the mice were euthanized and the tumors were dissected and were either frozen in RNAlater (Qiagen) or preserved in 4% paraformaldehyde. The tumor tissues were embedded in paraffin.

### RNA sequencing

D458 and HDMB03 cells were treated in duplicate with DMSO or CBL0137 (1 µM) for 24 h. Total RNA was extracted using TRIzol reagent. SmartQuerier Biotech (Shanghai, China) performed the RNA sequencing. STAR (v2.5.3a) was used to map the RNAseq reads to the hg38 reference genome. Moreover, cufflinks was used to calculate the values of FPKM (fragments per kilobase million) for each gene. Differential expression analysis was performed using DESeq2 (v1.20.0). Gene ontology, KEGG pathway and Hallmark analyses were carried out using the Investigate Gene Sets tool on the website http://software.broadinstitute.org/gsea/msigdb/annotate.jsp. Gene set enrichment analysis was performed according to the instructions on the website http://www.broadinstitute.org/gsea/index.jsp.

### Proliferation, apoptosis, and cell cycle assay

The Click-iT EdU Alexa Fluor 647 Flow Cytometry Assay Kit was used to measure cell proliferation. FITC Annexin V Apoptosis Detection Kit (556547, BD Biosciences) was used to analyse apoptosis. Cell cycle analysis was carried out using the cell cycle staining kit (CCS012, Metasciences). BD Fortessa FACS machine was used to analyse fluorescence-activated cell sorting (FACS), and FlowJo software was used to analyse the data.

### Cell viability assays

We seeded 5000 cells in 96-well plates in serial dilutions of CBL0137 in triplicate to assess the cellular responsiveness to CBL0137. Then, we measured cell viability by assessing ATP content 72 h later by using CellTiter-Glo (G7573, Promega), according to the manufacturer’s instructions. Nonlinear dose response curves were applied by using GraphPad Prism. MB cells were infected with lentiviral plasmids encoding shRNA to assess the dependence of cells on SSRP1. After 96 h, 5 × 10^3^ cells in each well of 96-well plates were cultured in the media containing puromycin (1 µg/ml). Finally, cell viability was measured in triplicate by assessing the ATP content using CellTiter-Glo (Promega) and the results were normalized to the baseline.

### H&E staining and immunohistochemical (IHC) staining

After 28 days of treatment in the intracranial orthotopic model, two mice from each group received ice-cold PBS and 4% formaldehyde via intracardiac perfusions. H&E staining for paraffin-embedded tissue sections was carried out by Runnerbio biotech. Comp. (Shanghai, China). IHC was performed with the following primary antibodies: Ki67 (ab15580, Abcam), cleaved caspase-3 (9664, CST) and MYC (5605S, CST).

### ChIP-qPCR assay

HDMB03 cells were cross-linked with 1% paraformaldehyde for 8 min at room temperature and quenched with 0.125 M glycine. HDMB03 cells were re-suspended in MNase reaction buffer plus MNase (M0247S, BioLabs) at 37 °C for 10 min. Digestion was terminated by the addition of EGTA. Chromatin was sonicated at 4 °C for 250 s with 20 s ultrasonication at 30 s intervals (Active Motif), and the resulting chromatin was immunoprecipitated at 4 °C for 12 h with the antibody against SSRP1 (13421S, CST) or IgG (2729S, CST). Antibody-chromatin complexes were pulled-down using Pierce ChIP-grade Protein A/G Magnetic Beads (26162, Thermo), and then washed and eluted by elution buffer. After cross-link reversal and proteinase K treatment, immunoprecipitated DNA was extracted with PCR Purification Kit (Vazyme Biotech). The DNA fragments were further analysed by real-time quantitative PCR using the primers as listed in Supplementary Table S1.

### Statistical analyses

GraphPad was used to analyse the experimental data which were presented in the form of mean ± standard deviation (SD). Two-tailed Student’s *t*-test was used to compare the differences between two groups, and log-rank test was used in the survival analysis. A *P* value less than 0.05 was regarded as statistically significant (*P < 0.05, **P < 0.01, and ***P < 0.001).

## Results

### Identification of SSRP1 as the top drug candidate of MYCamp-G3-MB within epigenetic modulators

**Fig. 1:**
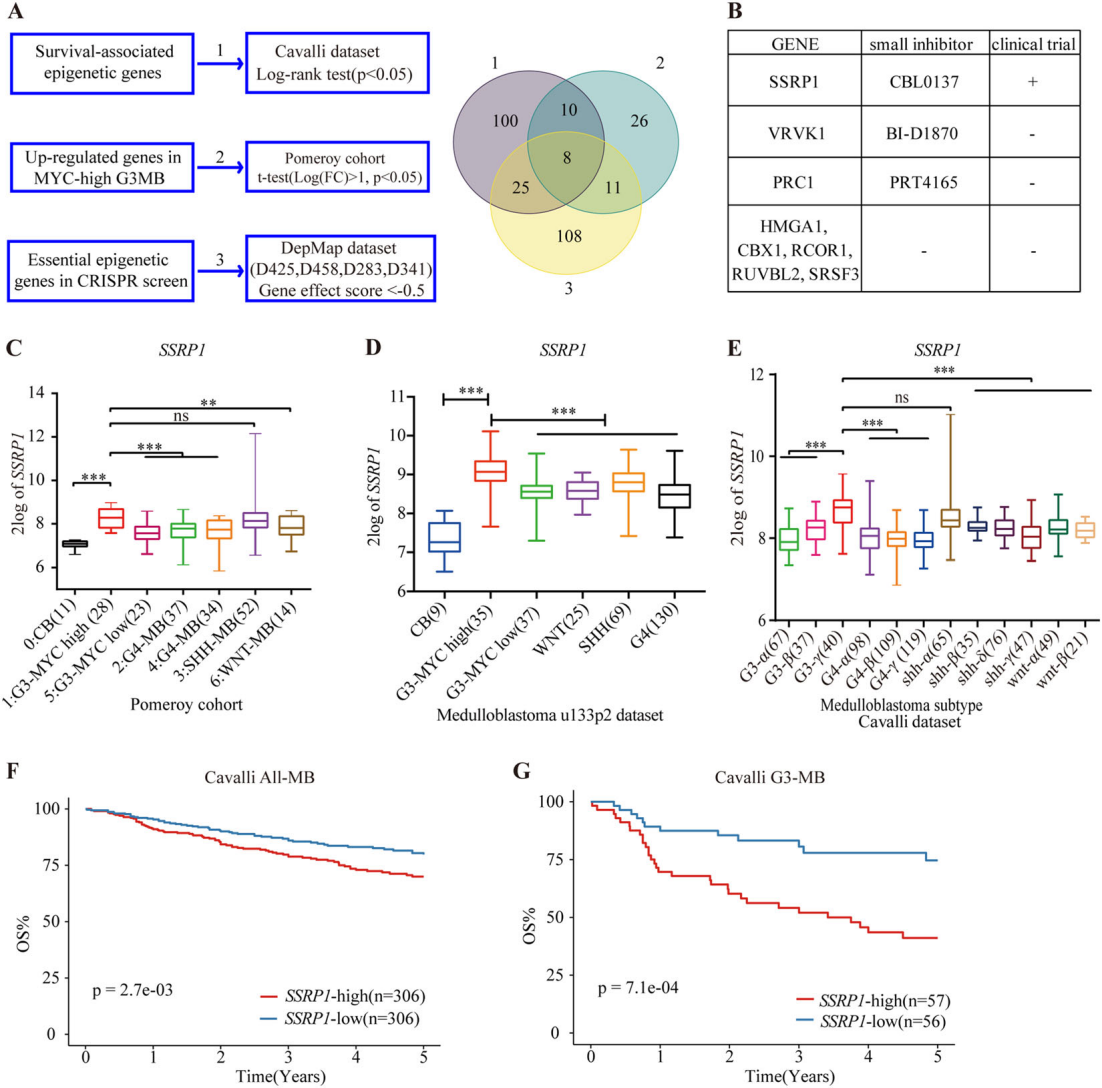
Integrated screen establishes SSRP1 as the top drug candidate and a prognostic marker. **A** Flowchart of bioinformatics analysis. **B** Genes with inhibitor were chosen for further validation. **C** Box plots of SSRP1 expression levels in different subgroups of medulloblastoma and normal cerebellum (CB) from the Pomeroy dataset. **D** Box plots of SSRP1 expression levels in medulloblastoma and normal cerebellum from the u133p2 dataset. **E** Box plots of SSRP1 expression levels in various subgroups of medulloblastoma from the Cavalli dataset. **F**, **G** Kaplan–Meier plots that show the OS rate in Cavalli dataset of all MB patients and group three MB patients. (*P < 0.05, **P < 0.01, and ***P < 0.001).

To systemically identify drug target candidates of epigenetic therapy in MYCamp-G3-MB, we firstly compiled a list of 870 epigenetic genes^21,22^ and performed bioinformatic analyses as follows: (1) identifying tumor-essential epigenetic genes (corrected CERES score < −0.5) from public available genome-scale CRISPR–Cas9 screening data of four *MYC* amplified or upregulated G3-MB cell lines (D283, D341, D425, and D458) from DepMap project (https://depmap.org/portal/); (2) identifying significantly upregulated epigenetic genes of MYCamp-G3-MB versus normal cerebellum (log2 fold change > 1, FDR < 0.05) from published medulloblastoma dataset (R2: Pomeroy dataset^15^); (3) identifying survival-associated epigenetic genes whose higher transcript levels are correlated with worse prognosis in published medulloblastoma dataset (log-rank *P* < 0.05, Cavalli dataset^19^) (Fig. 1A). Eight epigenetic genes (*CBX1*, *HMGA1*, *PRC1*, *RCOR1*, *RUVBL2*, *SRSF3*, *SSRP1*, and *VRK1*) were found to meet all above-mentioned criteria, whereas only *SSRP1* has targeted drug tested in human clinical trial and its role in tumorigenesis or cancer therapy has not been reported in MYCamp-G3-MB before (Fig. 1B). Thus, we chose *SSRP1* for further investigation.

SSRP1 forms a histone–chaperone FACT complex with its partner SPT16, and plays critical roles in regulating DNA replication, transcription, and repair of cancer cells^12,14,23,24^. Intriguingly, even though both FACT subunits coding genes are highly cancer-dependent in all the four *MYC* amplified or upregulated G3-MB cell lines, only *SSRP1* but not *SUPT16H* (the coding gene of SPT16) is significantly upregulated in MYCamp-G3-MB compared to normal cerebellum or most of the rest MB subtypes (MYClow-G3-MB, WNT-MB, G4-MB) across multiple published MB datasets (Pomeroy dataset, u133p2 dataset and Cavalli dataset, detailed in “Materials and methods” section) (Fig. 1C–E, and Figs. S1, S2) and its higher expression is associated with worse prognosis in all MB patients or G3-MB patients only (Fig. 1F, G).

**Fig. 2:**
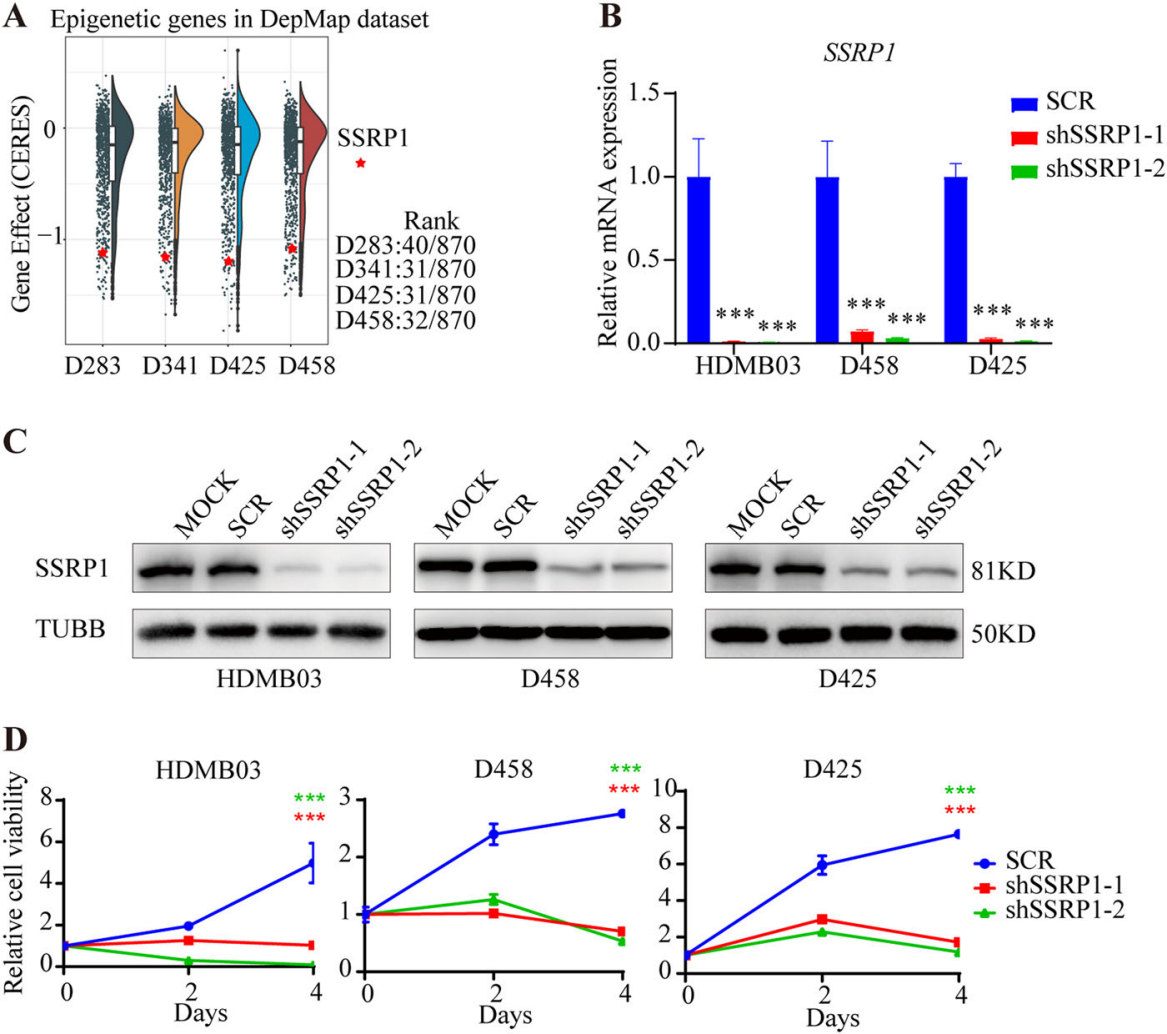
Targeted knockdown of SSRP1 using CRISPR-Cas9 or RNA interference attenuates the growth of medulloblastoma. **A** Boxplot of CRISPR gene effect scores in the MB cell lines. **B**, **C** RT-qPCR and western blot analyses were performed to assess the efficiency of shRNA SSRP1 knockdown. **D** Cell growth of the medulloblastoma cells infected with shRNA lentivirus was measured at days 0, 2, and 4. (*P < 0.05, **P < 0.01, ***P < 0.001).

To further verify the essential role of *SSRP1* in MYCamp-G3-MB as measured by functional genomic screening (Fig. 2A), we tested the inhibitory effects of *SSRP1* knockdown on cell viability in three MYCamp-G3-MB lines (HDMB03, D458, and D425). The knockdown efficiency of two individual shRNA against *SSRP1* were confirmed by both RT-qPCR and WB assays (Fig. 2B, C). Moreover, cell viability measurement results showed that depletion of SSRP1 resulted in substantial suppression of cell growth in all tested MYCamp-G3-MB lines in vitro (Fig. 2D).

### FACT antagonist CBL0137 is effective in inhibiting MYCamp-G3-MB in vitro

**Fig. 3:**
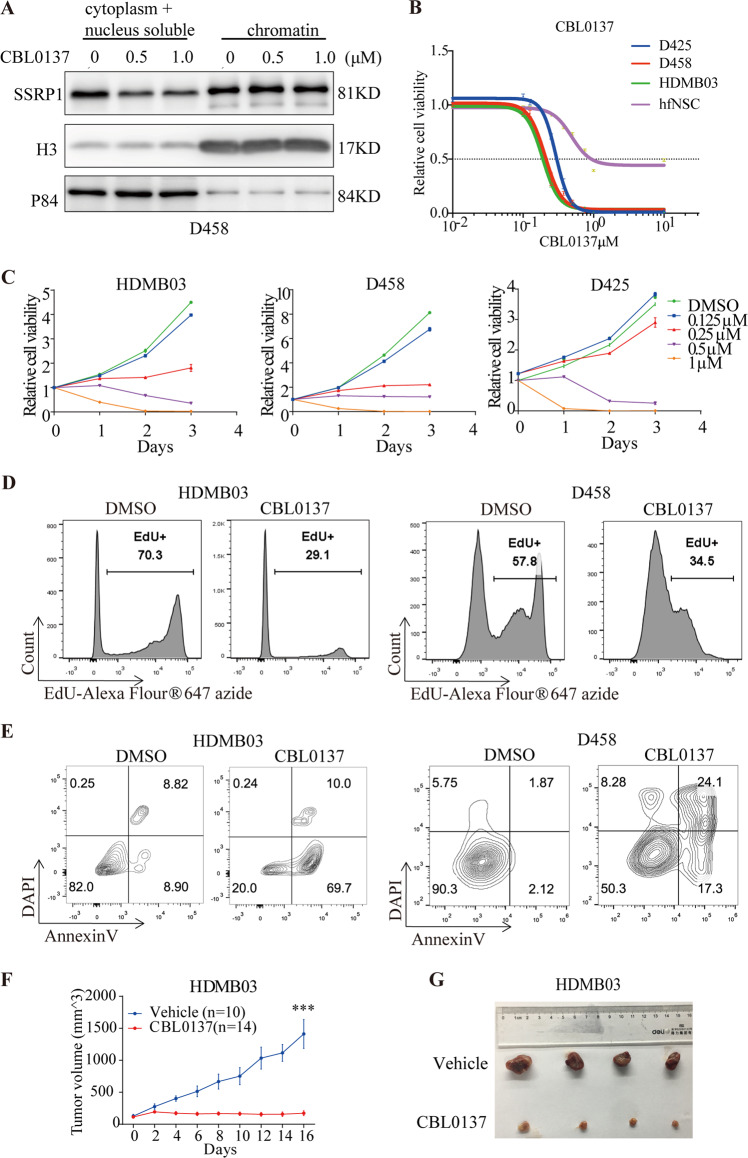
CBL0137 functions to inhibit the growth of MYC-amplified medulloblastoma cells. **A** Western blot analysis of SSRP1 protein level in cytoplasm, nucleus soluble, and chromatin bound fractions. **B** Dose-response curves of MYC-amplified medulloblastoma lines and control cell line hfNSC (human fetal neural stem cell) to CBL0137. **C** MYC-amplified medulloblastoma cell lines were treated with CBL0137 or DMSO. Post-treatment cell viabilities were monitored by CellTiter-Glo at days 1, 2, and 3 and were normalized to the value of day 0. **D** Proliferation of MYC-amplified medulloblastoma cells after the treatment with DMSO or 1 μM CBL0137 for 24 h was analysed using EdU incorporation FACS assays. **E** Apoptosis analyses of MYC-amplified medulloblastoma cells treated with DMSO or CBL0137 for 48 h by Annexin-V staining assay. **F** Tumor growth curves of subcutaneous xenograft HDMB03 tumor model. Nude mice with HDMB03 flank xenografts (each mouse carried two xenografts) were treated with CBL0137 at the dose of 70 mg/kg i.v.; q4d or cyclodextrin as a control. Data are shown as the mean ± SEM. **G** Images of the nude mice with subcutaneous xenograft HDMB03 tumors from vehicle group and CBL0137 treatment group.

As curaxin CBL0137, the FACT-targeted small-molecule drug, has already entered early-phase human clinical trials for cancer therapy, we aimed to test its efficacy of treating preclinical models of MYCamp-G3-MB. Firstly, we confirmed the quality of CBL0137 compound we used by measuring its effect on subcellular distribution of SSRP1 protein. We treated D458 cells with CBL0137 at 0.5 or 1.0 µM for 24 h and conducted cellular fractionation. In line with what was previously reported in other tumor types^25^, WB analysis results showed that CBL0137 sequestered SSRP1 into chromatin-bound fraction of MYCamp-G3-MB cells (Fig. 3A). Next, increasing concentrations of CBL0137 were used to treat three MYCamp-G3-MB lines and CellTiter-Glo assay was used to measure its effect on their growth in vitro. A human normal neural stem cell line hfNSC was also treated in parallel as control. As shown in Fig. 3B, C, CBL0137 treatment induced halted growth of D425, D458, and HDMB03 cells in a dose-dependent and time-dependent manner, and hfNSC line was much less sensitive to CBL0137 in comparison to the three MYCamp-G3-MB lines. Moreover, we showed that CBL0137 could dramatically suppress proliferation and induce apoptosis when treating MYCamp-G3-MB cells in vitro (Fig. 3D, E) while barely affected the control hfNSC cells (Fig. S3A, B).

### CBL0137 suppresses growth of MYCamp-G3-MB in vivo

To further evaluate the in vivo therapeutic efficacy of CBL0137, we firstly performed systemic CBL0137 treatment on two subcutaneous xenograft models of MYCamp-G3-MB, HDMB03 and D425. Our results showed that intravenous injection of CBL0137 at 70 mg/kg every four days could significantly disrupt the growth of both subcutaneous xenograft models (Figs. 3F,G and S4A, B). The measurements of Ki-67 and cleaved caspase-3 (CC3) levels in CBL0137 or vehicle treated xenografted tumor tissues harvested at the end of treatment revealed, that CBL0137 also dramatically suppressed proliferation and induced apoptosis in vivo (Fig. S4C).

**Fig. 4:**
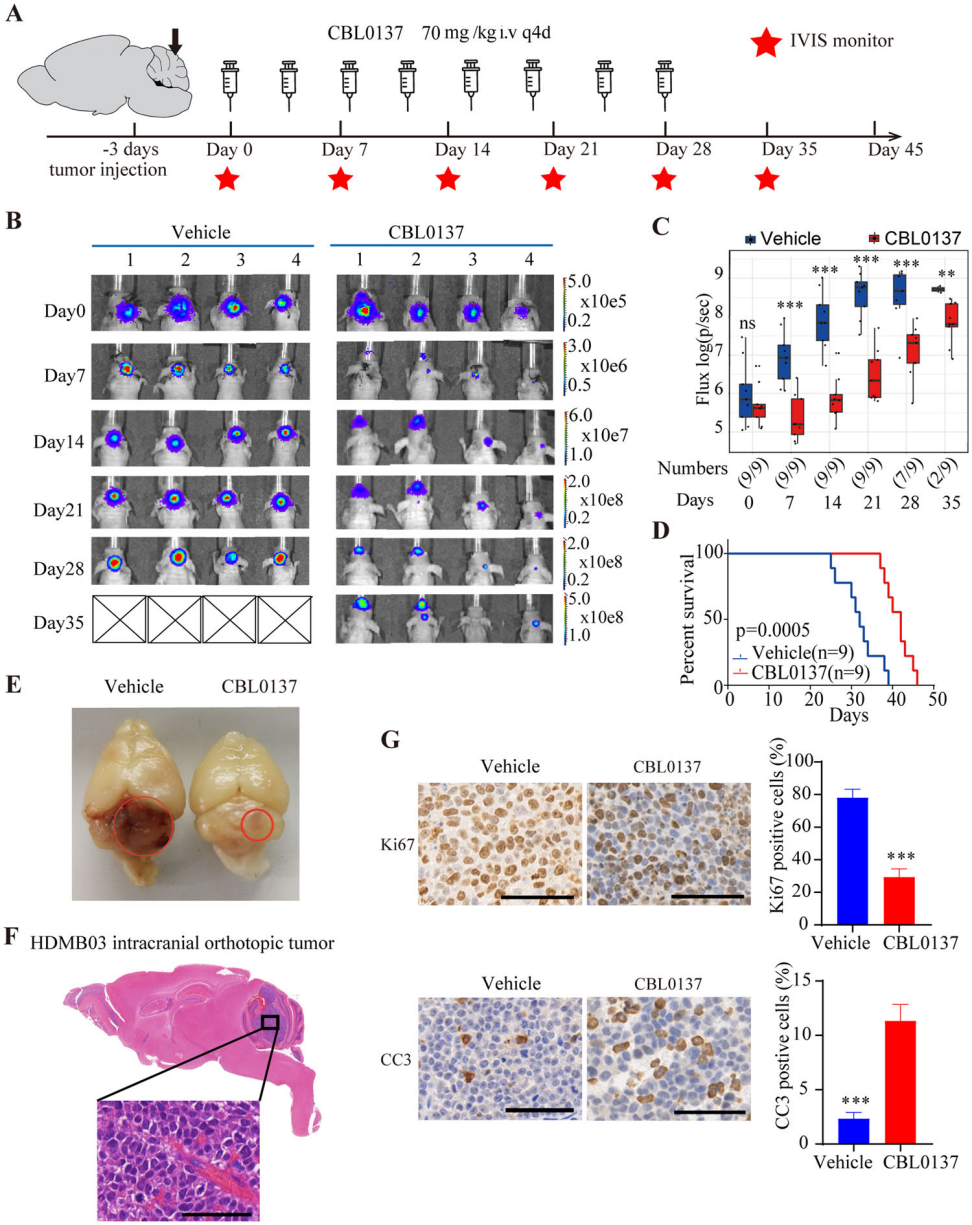
CBL0137 effectively inhibited the growth of MYC-amplified medulloblastoma in intracranial orthotopic tumor model. **A** Experimental protocol for the intracranial orthotopic medulloblastoma model. The HDMB03 MYC-amplified medulloblastoma cells were transduced with lentivirus containing a GFP-Luciferase construct to facilitate in vivo study. **B** IVIS images of HDMB03-bearing animals. Bioluminescence imaging was performed weekly since implantation (from day 0 to day 35). **C** Bioluminescence analysis of photon emission from mice which were orthotopically injected with HDMB03-luc cells. The differences in total flux (photon per second) were statistically significant between the CBL0137 group and the vehicle group. **D** Kaplan–Meier survival curves of mice. *P* value was determined by the log-rank test. **E** The animal from each group was euthanized on day 28. Gross appearance of the whole brain from the mouse in the vehicle group and the CBL0137 treatment group. The brain of the vehicle mouse showed enlarged tumor with hematoma; whereas the brain of CBL0137-treated mouse showed smaller tumors. **F** Up, HE stained of sagittal section of mouse brain euthanized on day 28 (50×). Down, detail of HE stained medulloblastoma section (scale bar: 50 µM). **G** Immunohistochemical staining of KI67 and CC3 (cleaved caspase-3) in vehicle group and CBL0137-treated intracranial tumors (scale bar: 50 µM). (*P < 0.05, **P < 0.01, ***P < 0.001).

Since the permeability of CBL0137 through blood-brain barrier has been demonstrated in glioblastoma models before^14,26^, we next tested its therapeutic efficacy in an orthotopic xenograft model of MYCamp-G3-MB. As shown in Fig. 4A, HDMB03 cells stably expressing luciferase were injected into the cerebellum of nude mice and treated with CBL0137 or vehicle 3 days after. CBL0137 were intravenously injected at 70 mg/kg every four days for 28 days, and IVIS imaging measurement was performed every seven days for six times. The survival of mice was monitored and recorded until all the tested mice died. Our IVIS imaging results showed that CBL0137 group of mice exhibited significantly reduced IVIS flux levels in comparison to vehicle group, indicating CBL0137 treatment substantially suppressed intracranial tumor growth of MYCamp-G3-MB (Fig. 4B, C). In line with this finding, CBL0137-treated group of mice survived significantly longer than vehicle group of mice (Fig. 4D). One mouse from each group were sacrificed at day 28 of treatment after the final injection and their brains were harvested. As shown in Fig. 4E, a much bigger and severer tumor was clearly seen at the cerebellum region of the mouse from vehicle group compared to the CBL0137-treated one. HE staining data confirmed the histology of large cell medulloblastoma with prominent nuclei (Fig. 4F) and IHC analyses of Ki67 and CC3 revealed CBL0137 disrupted the growth of orthotopic xenograft model of MYCamp-G3-MB through suppressing proliferation and inducing apoptosis in vivo (Fig. 4G).

### Transcriptome analyses demonstrate CBL0137 preferentially inhibits cell cycle and DNA repair in MYCamp-G3-MB

**Fig. 5:**
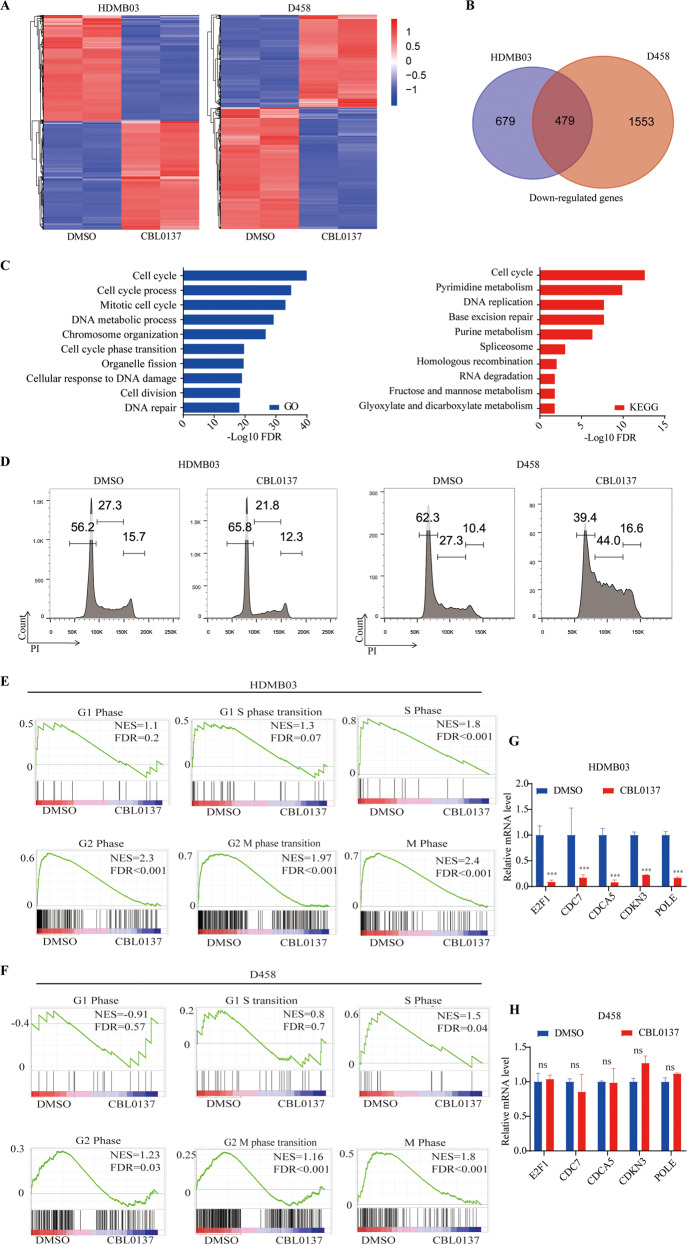
CBL0137 causes cell cycle arrest and suppresses cell cycle-associated genes expression. **A** Heatmap of relative gene expression levels of all mRNA transcripts in HDMB03 and D458 cells treated with CBL0137(1 µM) vs. DMSO. **B** Genes of the differentially downregulated expression in the CBL0137 treated HDMB03 and D458 cells were plotted as the Venn diagram to display the commonly downregulated genes. **C** The top pathways obtained from gene ontology and KEGG pathway analysis affected by CBL0137 treatment in D458 and HDMB03 cell lines. Bars represent significance of gene enrichment for a given pathway on a –log10(*P* value) scale. **D** FACS cell cycle analyses of medulloblastoma cells exposed to DMSO or CBL0137 for 24 h. **E**, **F** Enrichment plots of genes in cell cycle gene sets in DMSO-treated cells and CBL0137-treated cells. **G**, **H** G1 phase-associated genes expression in the MYC-amplified medulloblastoma cells which were treated with CBL0137 or DMSO was measured using RT-qPCR. (*P < 0.05, **P < 0.01, and ***P < 0.001).

To better understand CBL0137’s therapeutic effects against MYCamp-G3-MB, we performed transcriptomic analyses of HDMB03 and D458 cells treated with 1 µM CBL0137 or DMSO for 24 h. Our results showed that 8596 and 9225 significantly expressing genes (FPKM > 0.2) were detected by RNAseq and 1158 and 2032 genes were significantly downregulated by CBL0137 (log2 fold change < −1, FDR < 0.05) in HDMB03 and D458, respectively (Fig. 5A). Moreover, there were 479 shared significantly downregulated genes between the two lines (Fig. 5B), and GO and KEGG pathway analyses showed that these genes were enriched of cell cycle and DNA repair related biological processes (Fig. 5C).

Next, we performed cell cycle FACS staining experiments to verify the inhibitory effect of CBL0137 on cell cycle progression of MYCamp-G3-MB cells. Intriguingly, our data showed that HDMB03 and D458 responded differently to CBL0137 induced cell cycle arrest: G1 phase arrest in HDMB03 and S/G2-M phase arrest in D458 (Fig. 5D). To dissect the underlying mechanism of the response difference, gene set enrichment analysis (GSEA) of RNAseq data of CBL0137 treated MYCamp-G3-MB cells with various cell cycle-related gene sets (G1 Phase, G1 S phase transition, S Phase, G2 Phase, G2 M phase transition, and M Phase) and further RT-qPCR validation of multiple G1 phase and G1-S phase transition regulatory genes (*E2F1*^27^, *CDC7*^28^, *CDCA5*^29^, *CDKN3*^30^, and *POLE*^31^) were performed. Both GSEA and RT-qPCR results showed that G1 phase and G1-S phase transition regulatory genes were significantly inhibited by CBL0137 only in HDMB03 cells but not D458 cells, suggesting the different cell cycle arrest outcomes might result from their different sensitivity to CBL0137 between the two MYCamp-G3-MB lines (Fig. 5E–H).

### CBL0137 suppresses transcription of *MYC* and *NEUROD1* in MYCamp-G3-MB via depleting FACT complex from their promoter regions

**Fig. 6:**
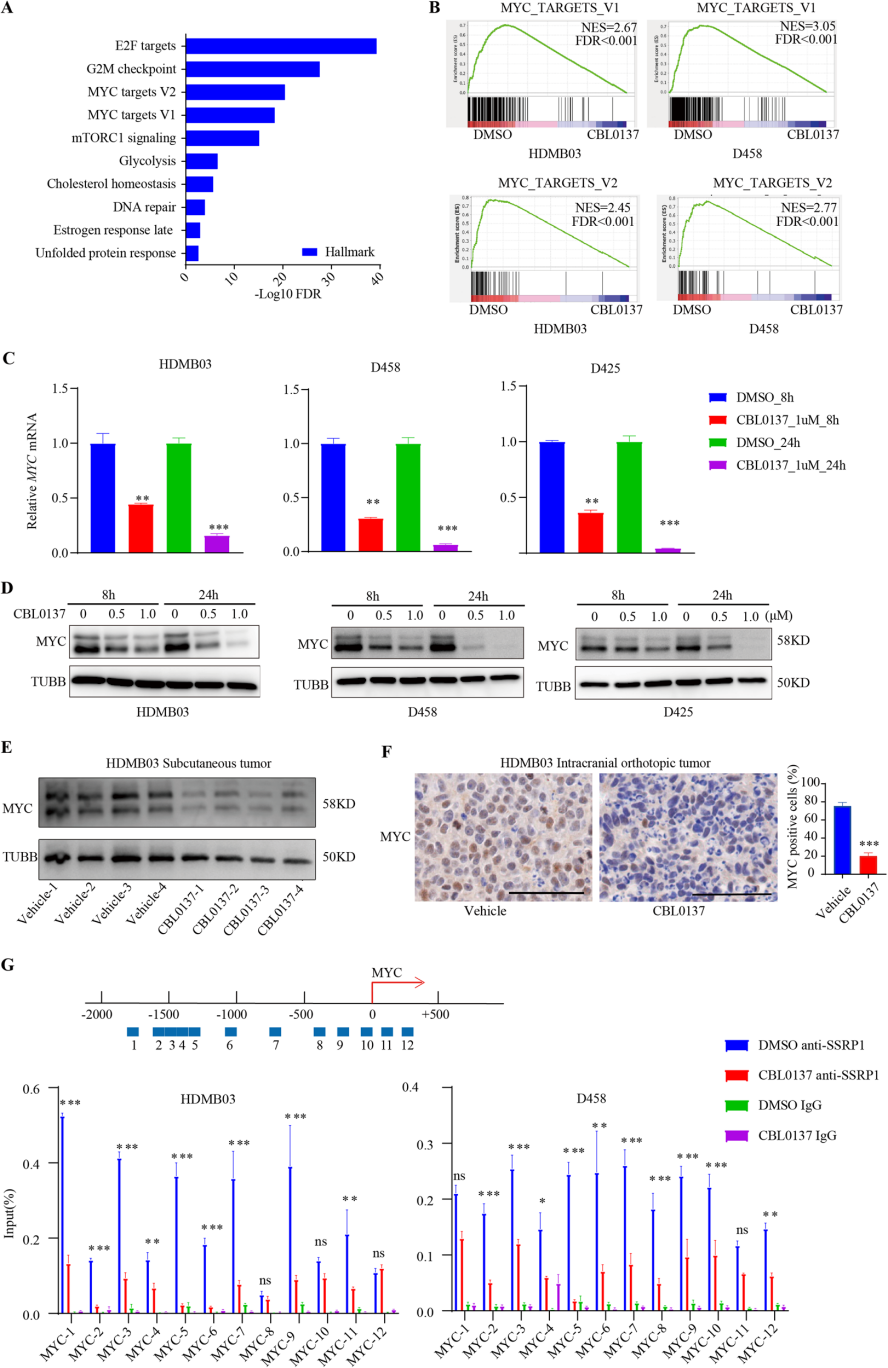
CBL0137 suppresses MYC and MYC-dependent transcription. **A** The top pathways obtained from hallmark pathway analysis affected by CBL0137 treatment in D458 and HDMB03 cell lines. Bars represent significance of gene enrichment for a given pathway on a –log10(*P* value) scale. **B** Enrichment plots from GSEA for the MYC_TARGETS gene sets in medulloblastoma cells which were treated with DMSO or CBL0137. **C** *MYC* expression in the MYC-amplified medulloblastoma cells treated with CBL0137 or DMSO was investigated using RT-qPCR. (*P < 0.05, **P < 0.01, and ***P < 0.001). **D** MYC expression in the MYC-amplified medulloblastoma cells treated with CBL0137 or DMSO was investigated using immunoblotting. **E** Immunoblotting analyses of MYC protein expression in the xenograft tumors derived from control and CBL0137-treated cells. **F** Immunohistochemical staining of MYC in vehicle or CBL0137-treated intracranial orthotopic tumors (scale bar: 50 µM). **G** ChIP-qPCR analysis of SSRP1 levels at MYC in MB cells treated with DMSO or CBL0137. The 12 sites at the promoter region of MYC ware arranged up the histogram. (*P < 0.05, **P < 0.01, and ***P < 0.001).

Notably, RNAseq analyses revealed that *MYC*, the well-established oncogenic driver and essential cancer-dependency of MYCamp-G3-MB^10^, was within the 479 shared significantly downregulated genes between the two MYCamp-G3-MB lines and it ranks 579th in HDMB03 and 211th in D458 among all detected genes according to the fold change of gene downregulation. Moreover, when Hallmark gene sets were tested for enrichment in the shared significantly downregulated genes, two MYC target gene sets (MYC_TARGETS_V1 and MYC_TARGETS_V2) were found to be 3rd and 4th enriched ones (Fig. 6A). Alternatively, GSEA results also confirmed that these two MYC target gene sets were significantly suppressed by CBL0137 in both MYCamp-G3-MB lines (Fig. 6B).

Next, the downregulation of *MYC* by CBL0137 in vitro at both RNA and protein levels were verified by RT-qPCR and WB in three MYCamp-G3-MB lines (HDMB03, D458, and D425). Our results showed that CBL0137 could induce a dramatic decrease of *MYC* transcript at as early as 8 h post treatment in all tested lines (Fig. 6C, D). The downregulation of MYC was also confirmed in vivo in both CBL0137-treated subcutaneous and intracranial xenograft models of MYCamp-G3-MB by WB and IHC assays respectively (Fig. 6E, F). Furthermore, we performed ChIP-qPCR analyses of DMSO-treated or CBL0137-treated HDMB03 and D458 cells and our results showed that SSRP1 was able to bind to *MYC* promoter region and this binding was significantly disrupted by CBL0137 treatment, indicating CBL0137 suppressed *MYC* transcription through depleting FACT complex from its promoter (Fig. 6G).

**Fig. 7:**
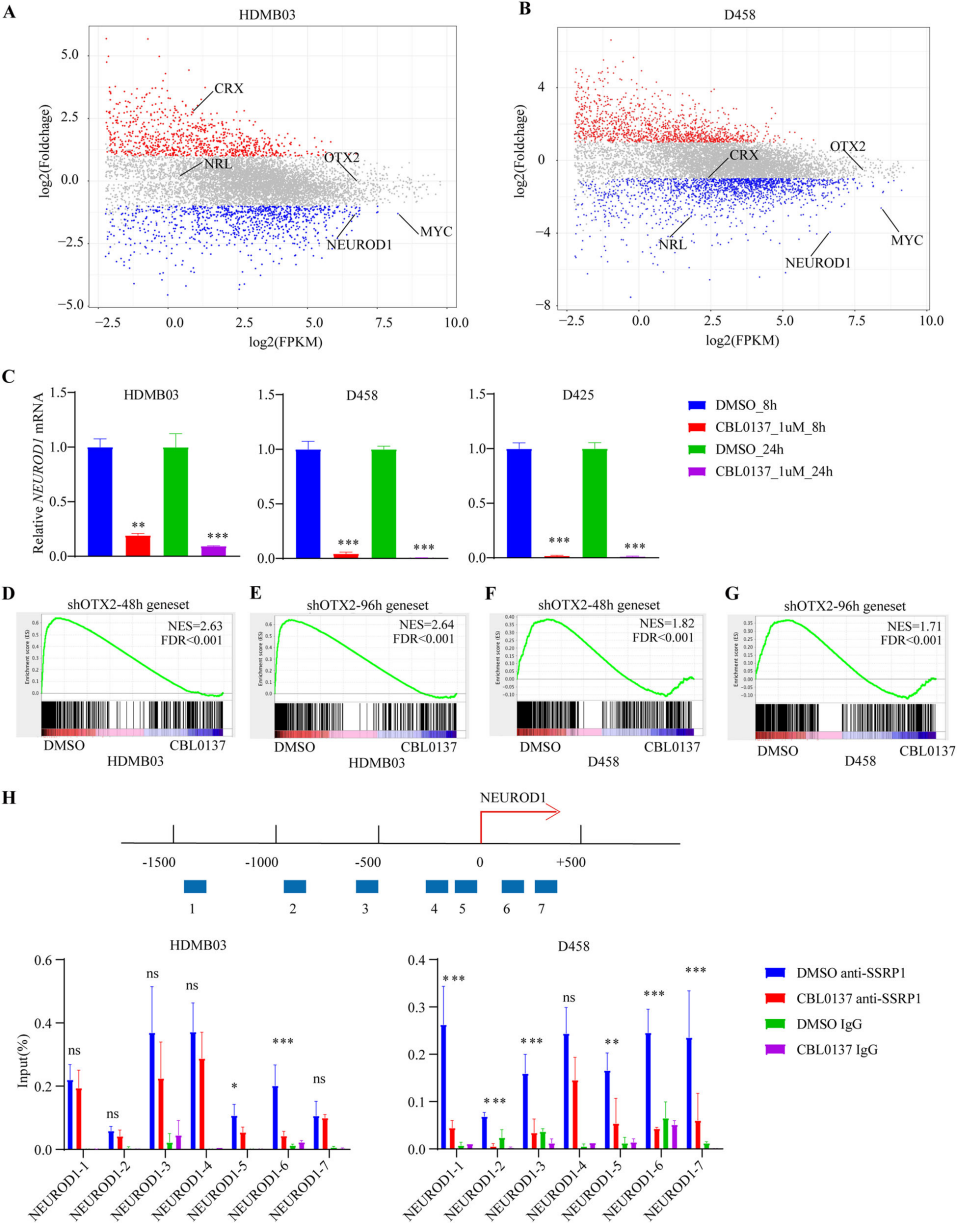
CBL0137 decreases the transcription of NEUROD1 and OTX2-associated genes. **A**, **B** Volcano plot of the normalized expression of genes in MYC-amplified medulloblastoma cells after CBL0137 treatment. Red points represent genes with *P*-value <0.05. **C** *NEUROD1* expression in the MYC-amplified medulloblastoma cells which were treated with CBL0137 or DMSO was measured using RT-qPCR. **D**–**G** Enrichment plots of genes in shOTX2_48 h and shOTX2_96 h gene sets in DMSO-treated cells and CBL0137-treated cells. **H** ChIP-qPCR analysis of SSRP1 levels at MYC in MB cells treated with DMSO or CBL0137. The seven sites at the promoter region of NEUROD1 ware arranged up the histogram. (*P < 0.05, **P < 0.01, and ***P < 0.001).

The finding of *MYC* as a critical target gene of FACT complex in MYCamp-G3-MB prompted us to examine potential roles of other well-established oncogenic driver transcription factors, such as OTX2^32,33^, NEUROD1^34^, CRX^35^, and NRL^35^, in FACT-related oncogenesis and targeted therapy. As shown in Fig. 7A, B, in addition to *MYC*, *NEUROD1* was also significantly downregulated by CBL0137 in both HDMB03 and D458. The RNAseq data was confirmed by RT-qPCR validation in three MYCamp-G3-MB lines (Fig. 7C). A recent study demonstrates that NEUROD1 exerts its oncogenic transcription factor role in maintaining tumor enhancer landscape of G3-MB in cooperation with OTX2^34^. Thus, we built OTX2-NEUROD1 target gene sets of MYCamp-G3-MB consisted of significantly downregulated genes (log2 fold change < −1, FDR < 0.05) in D425 cells stably expressing shOTX2 for 48 or 96 h with previously published gene expression profile GSE22875^20^, and subjected them to GSEA test of RNAseq data of DMSO or CBL0137 treated HDMB03 or D458 cells. The results showed that CBL0137 treatment also resulted in significant downregulation of the two OTX2-NEUROD1 target gene sets in both MYCamp-G3-MB lines, indicating the suppression of their downstream oncogenic transcriptional programs by FACT inhibition as well (Fig. 7D–G). Lastly, ChIP-qPCR analyses revealed the binding of SSRP1 to *NEUROD1* promoter region as well as the significant disruption of this binding by CBL0137 (Fig. 7H), suggesting a similar targeting mechanism of FACT inhibition on *NEUROD1* as *MYC* (Fig. 6G).

## Discussion

In this study, we aimed to identify novel effective therapeutic strategy against MYCamp-G3-MB, which exhibits the worst prognosis of the most common malignant pediatric brain tumor. Recently along with the recognition of the critical role of epigenetic modulators in regulating tumorigenesis, invasiveness and response to conventional therapy, epigenetic-targeted therapy has been shown promising antitumor activity across multiple cancer types and many such small-molecule inhibitors or drugs have been developed or under-development^36–38^. In MYCamp-G3-MB, a few epigenetic modulators, such as BRD4, SETD8, and HDACs, have been shown to be effective therapeutic targets, indicating a promising new direction of therapy development^7–9^.

Here we reported SSRP1 as another promising drug target candidate for epigenetic therapy against MYCamp-G3-MB. *SSRP1* was initially identified by bioinformatic analyses and then functionally validated to meet the following criteria: (1) significantly upregulated in MYCamp-G3-MB compared to normal cerebellum; (2) significantly correlated with worse prognosis in MB; (3) exhibits crucial cancer-dependency in MYCamp-G3-MB cells. SSRP1 and SPT16 consist of the histone-chaperone FACT complex that plays important roles in regulating transcription, cell cycle and DNA repair in cancer cells^12,39,40^. Upregulation of either one of the two FACT genes could lead to FACT activation and is associated with more aggressiveness and worse prognosis in various cancer types^26,41^. In MYCamp-G3-MB, SPT16 does not fulfill the first two criteria despite exhibiting crucial cancer-dependency, whereas in another MYC family member driven malignant pediatric solid tumor, *MYCN*-amplified neuroblastoma (MYCNamp-NB), SPT16 is the major oncogenic player instead of SSRP1^42^.

In our study, SSRP1 was selected for further investigation also because of the availability of its targeted drug which possesses BBB penetrating capacity and has entered early-phase human clinical trials^14,26^. Thus, FACT-targeted curaxin drug CBL0137 was further tested on multiple MYCamp-G3-MB models in vitro and in vivo and consistently exhibited robust therapeutic effects, including an orthotopic intracranial xenograft model. RNAseq analyses of CBL0137-treated MYCamp-G3-MB cells showed that cell cycle and DNA repair were the top two targeted biological processes, proving its on-target inhibitory effects. Moreover, targeting DNA repair indicates the potential combinatory use of CBL0137 with conventional therapies in treating MYCamp-G3-MB as demonstrated in other cancers, such as MYCNamp-NB, glioblastoma, small cell lung cancer and pancreatic cancer^14,42–44^. The therapeutic effect of CBL0137 on MYCamp-G3-MB cells was further demonstrated by its inhibition on transcription of two well-described oncogenic transcription factors (TFs), *MYC* and *NEUROD1*, via depleting FACT complex from their promoter regions. However, it remains unknown why CBL0137 treatment selectively targets these two oncogenic TFs without affecting others including OTX2, CRX, and NRL. Further anti-SSRP1 ChIPseq analyses on DMSO-treated or CBL0137-treated MYCamp-G3-MB cells may help better understand this discrepancy.

Taken together, our study demonstrates that FACT-targeted curaxin drug CBL0137 works effectively in treating MYCamp-G3-MB preclinical models both in vitro and in vivo, providing evidence for initiating human clinical trials against this devastating form of MB in near future. Moreover, our study further supports that epigenetic-targeted therapy is a promising direction for identifying novel effective treatment for MYCamp-G3-MB, therefore, the other top candidates identified in our initial bioinformatic analyses are currently under active investigation to identify potential therapeutic targets for future drug development.

## Supplementary information

Supplemental figure legend

Figure S1

Figure S2

Figure S3

Figure S4

Supplementary table S1
