# Supplementary material for: Effective inhibition of MYC-amplified group 3 medulloblastoma by FACT-targeted curaxin drug CBL0137
Source: Cell Death Dis. 2020 Dec 2;11(12):1029. doi: 10.1038/s41419-020-03201-6 (PMC7710710; doi:10.1038/s41419-020-03201-6)
Supplement: Supplementary file 1 — Supplemental figure legend [file 41419_2020_3201_MOESM1_ESM.docx]

**Figure S1. MYC expression in primary medulloblastomas.**

(A) Box plots of *MYC* expression levels in different subgroups of medulloblastoma and normal cerebellum (CB) from the Pomeroy dataset. (B) Box plots of *MYC* expression levels in medulloblastoma and normal cerebellum from the u133p2 dataset.

**Figure S2. SUPT16H expression and correlation in primary medulloblastomas.**

(A) Box plots of *SUPT16H* expression levels in different subgroups of medulloblastoma and normal cerebellum (CB) from the Pomeroy dataset. (B) Box plots of *SUPT16H* expression levels in medulloblastoma and normal cerebellum from the u133p2 dataset. (C) Box plots of *SUPT16H* expression levels in various subgroups of medulloblastoma from the Cavalli dataset. (D, E) Kaplan-Meier plots that show the OS rate in Cavalli dataset of all MB patients and group 3 MB patients. (F) Boxplot of CRISPR gene dependency scores in the MB cell lines. (*P<0.05, **P<0.01, ***P<0.001).

**Figure S3. CBL0137 doesn’t affect apoptosis, cell proliferation and cell cycle in hfNSC cells.**

(A) Apoptosis analyses of hfNSCs treated with DMSO or CBL0137 for 48 hours by Annexin-V staining assay. (B) Proliferation of hfNSCs after the treatment with DMSO or 1 μM CBL0137 for 24h was analysed using EdU incorporation FACS assays. (C) FACS cell cycle analyses of hfNSCs exposed to DMSO or CBL0137 for 24 hours.

**Figure S4. CBL0137 effectively inhibited the growth of MYC-amplified medulloblastoma in subcutaneous tumour models.**

(A) Tumour growth curves of subcutaneous xenograft D425 tumour model. (B) Images of subcutaneous xenograft D425 tumours of control group and CBL0137 treatment groups. (C) Immunohistochemical staining of Ki67 and CC3 (cleaved caspase-3) in control group and CBL0137-treated HDMB03 subcutaneous tumours (scale bar: 50 µM). (*P<0.05, **P<0.01, ***P<0.001).
