# Supplementary figures and images for: Effective inhibition of MYC-amplified group 3 medulloblastoma by FACT-targeted curaxin drug CBL0137
Source: Cell Death Dis. 2020 Dec 2;11(12):1029. doi: 10.1038/s41419-020-03201-6 (PMC7710710; doi:10.1038/s41419-020-03201-6)

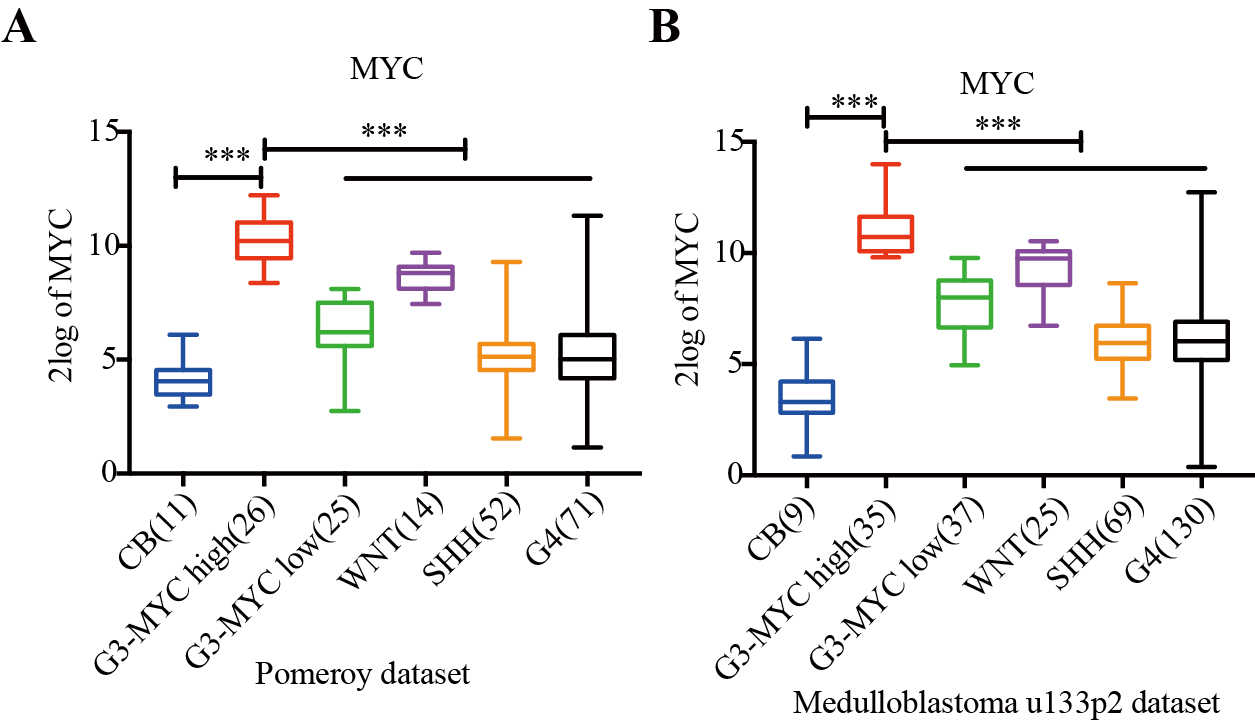

Supplement: Supplementary file 2 — Figure S1 [file 41419_2020_3201_MOESM2_ESM.tif]

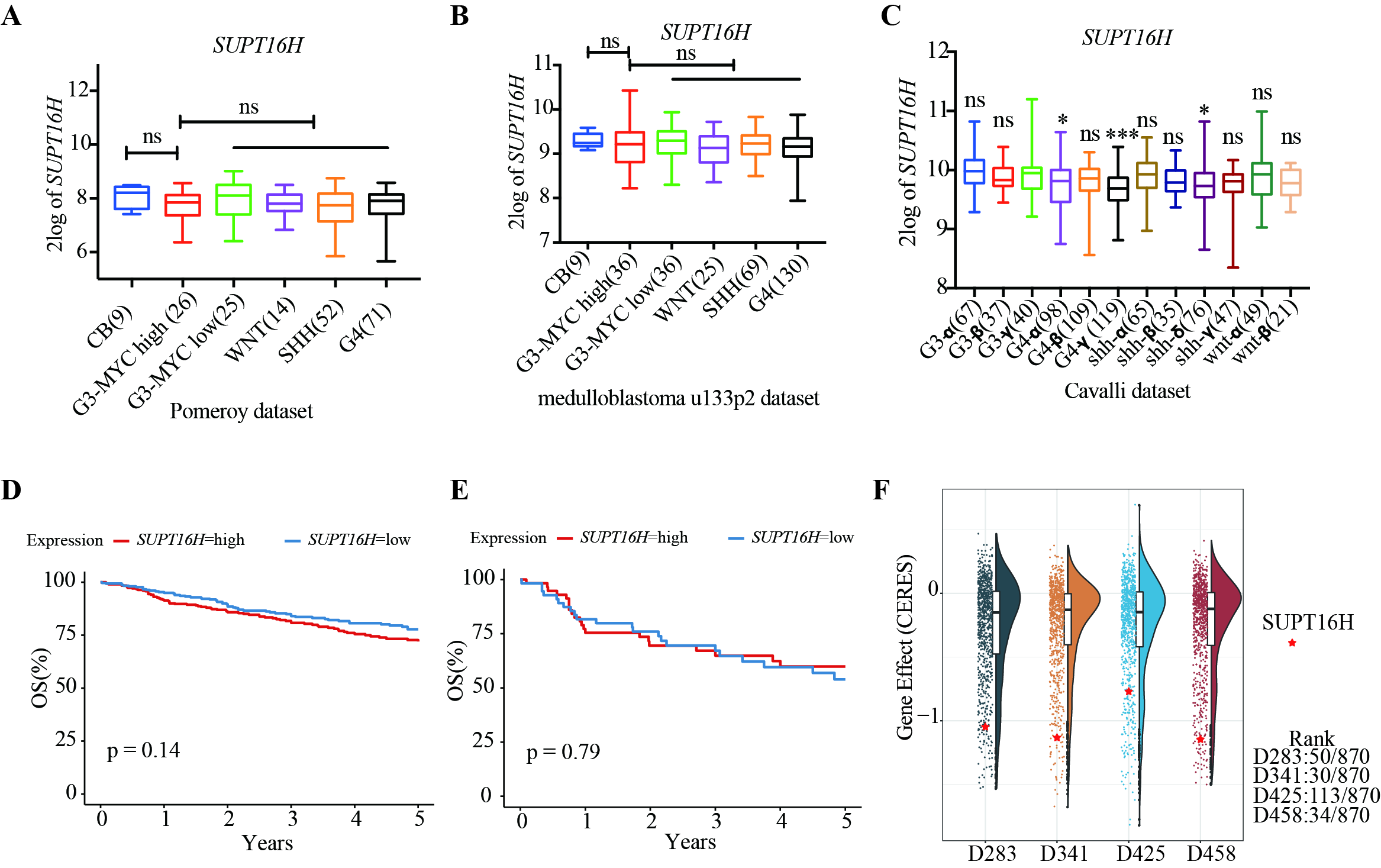

Supplement: Supplementary file 3 — Figure S2 [file 41419_2020_3201_MOESM3_ESM.tif]

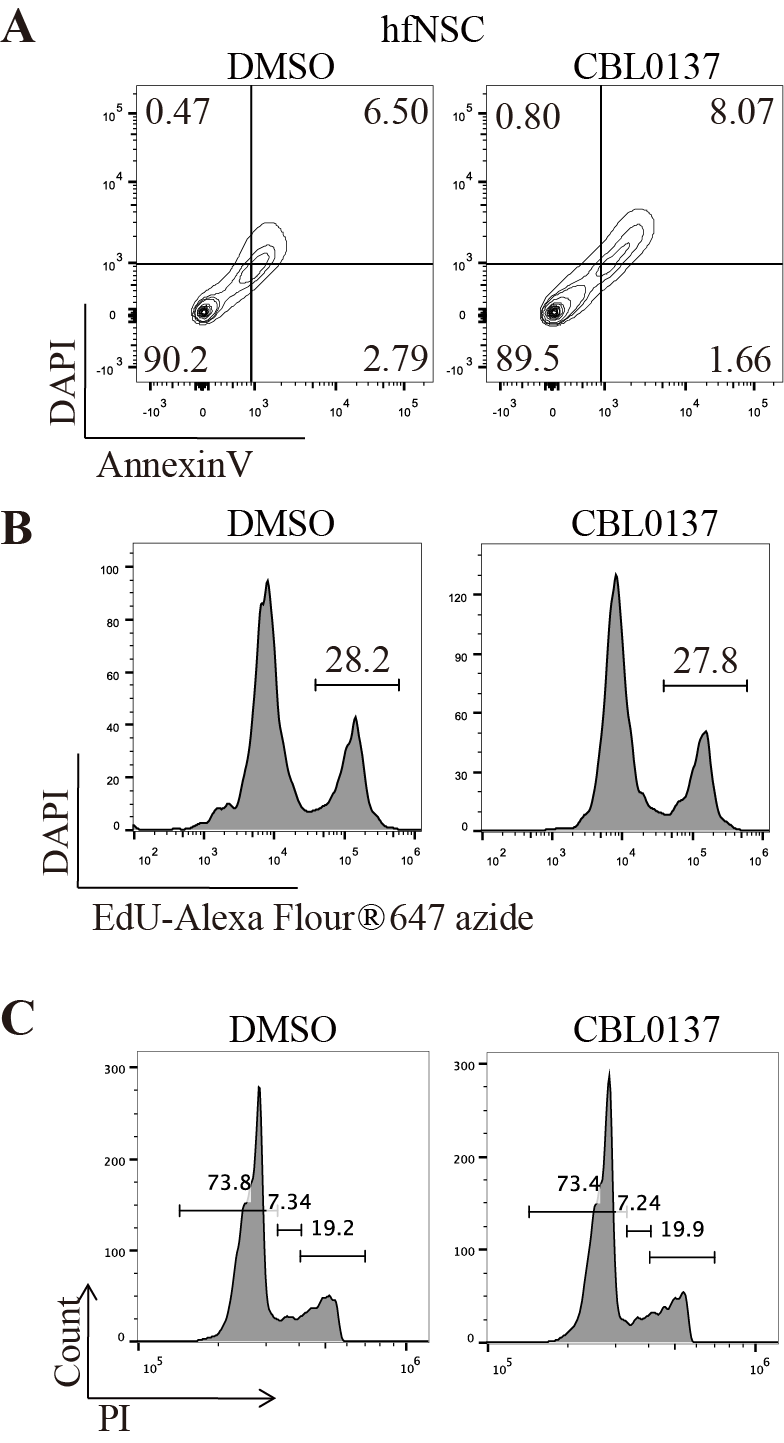

Supplement: Supplementary file 4 — Figure S3 [file 41419_2020_3201_MOESM4_ESM.tif]

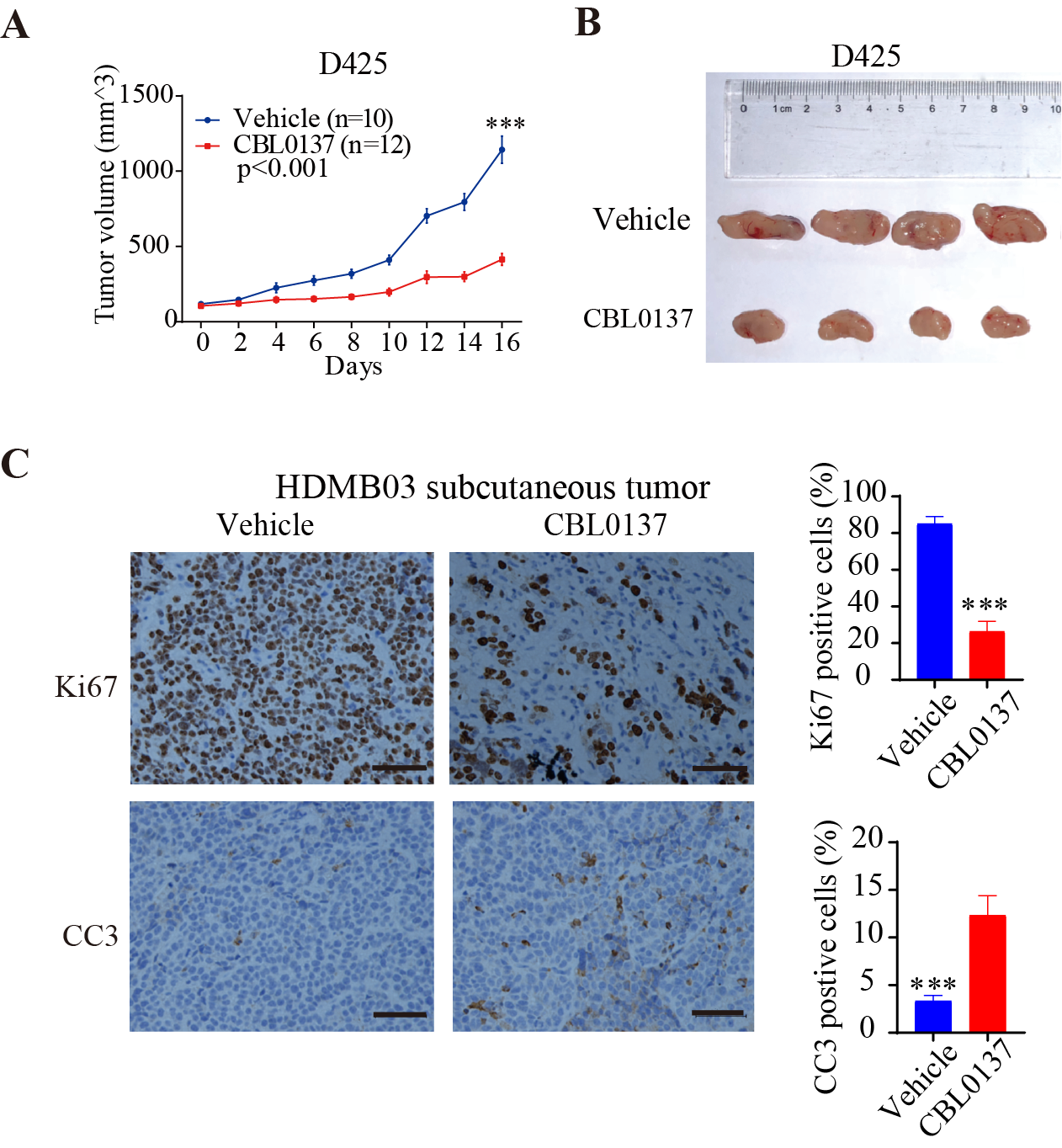

Supplement: Supplementary file 5 — Figure S4 [file 41419_2020_3201_MOESM5_ESM.tif]
